# Supplementary material for: Bioengineering of Escherichia coli Nissle 1917 for Production and Excretion of Spermidine, a Key Metabolite in Human Health
Source: Metabolites. 2022 Nov 2;12(11):1061. doi: 10.3390/metabo12111061 (PMC9697600; doi:10.3390/metabo12111061)
Supplement: Supplementary file 1 [file metabolites-12-01061-s001.zip › Supplementary Fig 1-1.pdf]

Supplementary Figure S1

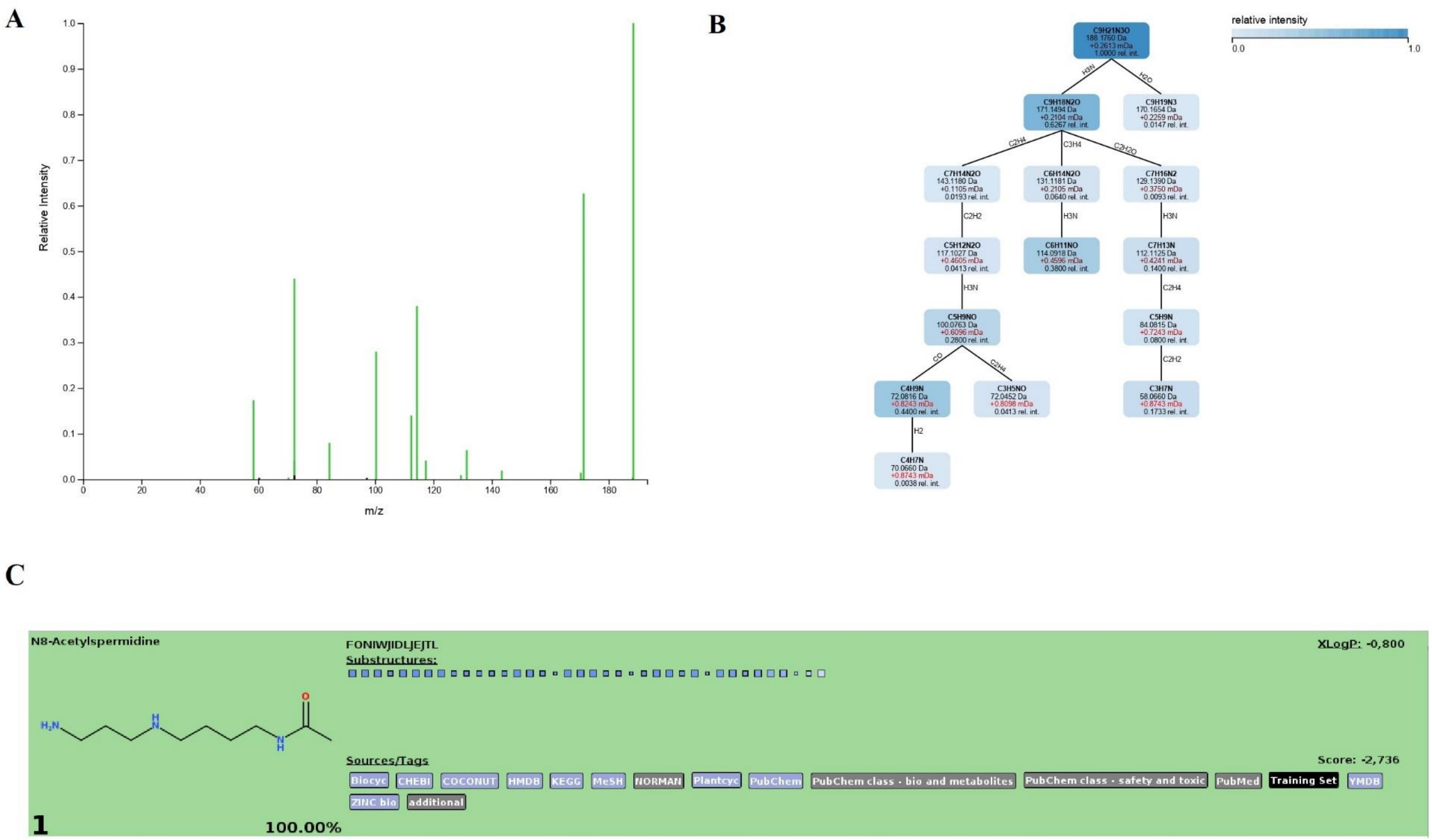

Supplementary Figure S1: Metabolite annotation of Acetylspermidine (precursor ion : 188.17 m/z) using SIRIUS and CSI:FingerID. (A) Fragmentation profile (MS/MS) of the precursor ion 188.17 m/z **(B)** Formula identification using SIRIUS: This step allows the prediction of the formula of the precursor ion based on its fragmentation profile (MS/MS). The precursor ion formula is predicted to be $C_9H_{21}N_3O$. **(C)** CSI:FingerID -Structure Database Search : The similarity of our molecular fingerprint is 100% for N8-Acetylspermidine**.**
