# Supplementary material for: Bioengineering of Escherichia coli Nissle 1917 for Production and Excretion of Spermidine, a Key Metabolite in Human Health
Source: Metabolites. 2022 Nov 2;12(11):1061. doi: 10.3390/metabo12111061 (PMC9697600; doi:10.3390/metabo12111061)
Supplement: Supplementary file 1 [file metabolites-12-01061-s001.zip › Supplementary Fig 3 (Ex1)-1.pdf]

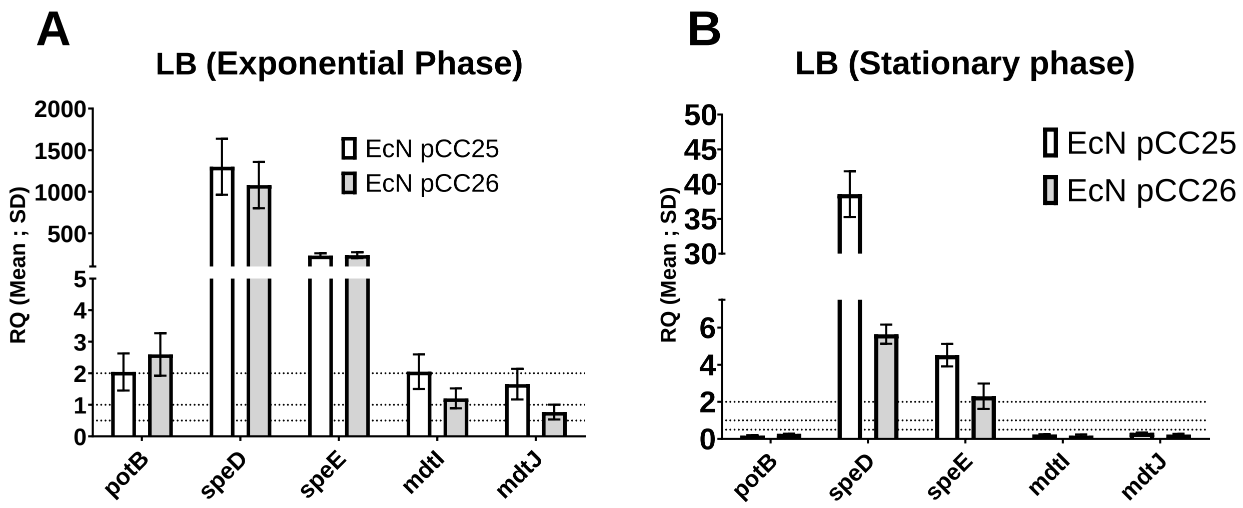


**Supplementary Figure S3.** Relative quantification of PAs related genes expression (*potB*; *mdtI*; *mdtJ*; *speD*; *speE*) in EcN, EcN pCC25 and EcN pCC26 in exponential phase **(A)** or stationary phase (**B**). Reference sample: EcN cultured in the same experimental conditions described in the material and method section. Endogenous control: rRNA-16S ribosomal RNA. All data shown represent the mean ± SD.

*Total RNA extraction and first strand cDNA synthesis*

Total RNA was extracted using NucleoSpin RNA, MiniKit (#740955.50-MACHEREY-NAGEL), as recommended by the manufacturer's protocol. RNA integrity was analyzed by agarose gel electrophoresis and its purity and concentration were calculated by measuring the optical density of the samples at 260 and 280 nm using a spectrophotometer.

For single strand cDNA synthesis, 1 µg of high-quality purified RNA was reverse transcribed in a 20 µL volume reaction using random hexamers and Super Script First-Strand Synthesis System For RT-qPCR (# 11904018-invitrogen), according to the manufacturer's instructions (Invitrogen).

*Real-time PCR assays*

The Real-time PCR assays were carried out in a Real-Time PCR System (Applied Biosystems) using the GoTaq qPCR Master Mix (#A6001-Promega), following the manufacturer's instructions. Reaction mixture consisted of 1 µL of diluted cDNA, 200 nM of each primer, CXR reference dye at final concentration of 300 nM and 1x Master Mix (2X) adjusted with nuclease free water to a final volume of 20µL.

The reactions were initially incubated for 2 minutes at 95 °C. After this pretreatment, reactions were subjected to the following thermal cycling conditions: 40 cycles of denaturation at 95 °C for 15 seconds and annealing/extension at 60 °C for 60 seconds. Finally, dissociation (melting) curve analyses were performed to check for nonspecific amplification and/or primer-dimers formation.

Primers used for this experiment:

| **Gene of Interest** | Primers used | Tm | %GC |
|---|---|---|---|
| *mdtI* | FwD : TTTAGTGCGCTTTCTCAGGC<br>Rev : GCAAGACCAGGCCAATCC | 55.31°C<br>55.11°C | 50.00%<br>61.11% |
| *mdtJ* | FwD : ACGCTGTCTATGAAATGGGC<br>Rev : TACCGATACCTTCCCACAGC | 54.77°C<br>55.26°C | 50.00%<br>55.00% |
| *potB* | FwD : TGGGCCTGTTCTATGTGTCC<br>Rev : GCAACATCAGGCCCATTACG | 55.73°C<br>56.09°C | 55.00%<br>55.00% |
| *speD* | FwD : AAGGCGCTGTATGACATGGT<br>Rev : CAGCGGTAATTTCCTGGCG | 56.12°C<br>55.92°C | 50.00%<br>57.89% |
| *speE* | FwD : CCCGGCGAAAGTCTGTTTAC<br>Rev : CGCTGAAGTAATGGCTGAGT | 55.71°C<br>54.44°C | 55.00%<br>50.00% |
| *16S* | FwD : GCTACAATGGCGCATACAAA<br>ReV : TTCATGGAGTCGAGTTGCAG | 56°<br>57° | 45.00%<br>50% |
